# AJUBA LIM Proteins Limit Hippo Activity in Proliferating Cells by Sequestering the Hippo Core Kinase Complex in the Cytosol

**DOI:** 10.1128/MCB.00136-16

**Published:** 2016-09-26

**Authors:** Radhika Jagannathan, Gregory V. Schimizzi, Kun Zhang, Andrew J. Loza, Norikazu Yabuta, Hitoshi Nojima, Gregory D. Longmore

**Affiliations:** a The ICCE Institute, Washington University, St. Louis, Missouri, USA; b Department of Medicine (Oncology), Washington University, St. Louis, Missouri, USA; c Department of Cell Biology and Physiology, Washington University, St. Louis, Missouri, USA; d Department of Biochemistry and Biophysics, Washington University, St. Louis, Missouri, USA; e Department of Molecular Genetics, Research Institute for Microbial Disease, Osaka University, Osaka, Japan

## Abstract

The Hippo pathway controls organ growth and is implicated in cancer development. Whether and how Hippo pathway activity is limited to sustain or initiate cell growth when needed is not understood. The members of the AJUBA family of LIM proteins are negative regulators of the Hippo pathway. In mammalian epithelial cells, we found that AJUBA LIM proteins limit Hippo regulation of YAP, in proliferating cells only, by sequestering a cytosolic Hippo kinase complex in which LATS kinase is inhibited. At the plasma membranes of growth-arrested cells, AJUBA LIM proteins do not inhibit or associate with the Hippo kinase complex. The ability of AJUBA LIM proteins to inhibit YAP regulation by Hippo and to associate with the kinase complex directly correlate with their capacity to limit Hippo signaling during Drosophila wing development. AJUBA LIM proteins did not influence YAP activity in response to cell-extrinsic or cell-intrinsic mechanical signals. Thus, AJUBA LIM proteins limit Hippo pathway activity in contexts where cell proliferation is needed.

## INTRODUCTION

Proliferating metazoan cells, upon formation of a complete organ *in vivo*, undergo growth arrest or cessation of proliferation, which is critical for the ultimate determination of organ size during development. The Hippo tumor suppressor pathway, which is highly conserved from Drosophila to humans, is a central signaling pathway controlling organ size during development by regulating cell apoptosis and proliferation. The Hippo pathway is also important for tissue regeneration and repair in response to injury in adult organisms, and its deregulation appears to contribute to both tumor development and suppression (1, 2).

At its core, the Hippo pathway is a kinase cascade. The Ste-20 kinases, MST1 and MST2 (Drosophila *Hpo*), along with the WW domain-containing scaffold protein WW45 (Drosophila *Sav*), bind to and phosphorylate the scaffolding proteins MOB1A/MOB1B (Drosophila *Mats*) (3). Phosphorylation of MOB1 enhances its association with the NDR kinases LATS1 and LATS2 (Drosophila *Wts*) and their phosphorylation and subsequent activation by MST kinases (4, 5). LATS1/LATS2 in turn phosphorylate the transcriptional coactivators YAP and TAZ (Drosophila *Yki*) at multiple sites, some of which lead to the sequestration of YAP/TAZ in the cytosol through an interaction with 14-3-3 proteins, while others target YAP/TAZ for degradation by the proteasome (4, 6). In the absence of LATS activation (i.e., the Hippo pathway is off), YAP and TAZ are predominantly nuclear, where they bind to the TEAD family of transcription factors and enhance the transcription of proproliferative and antiapoptotic genes, and cell proliferation ensues (7, 8).

The upstream signals activating the Hippo pathway, which leads to phosphorylation and inactivation of YAP and cessation of proliferation, are diverse and can involve distinct intracellular signaling cascades. They include changes in cell-cell contact, cell polarity, cell tension, anoikis, and hormonal signals (9–14). In particular, the Hippo pathway plays an important role in cell-cell contact inhibition of proliferation (CIP) (11). As epithelial cells come into contact with one another, they adhere to form a confluent sheet of cells, cell-cell adhesion activates the Hippo pathway that inhibits YAP, and proliferation slows (13). Further increase in cell density leads to cell compaction, the individual cell area decreases, and cell proliferation ceases altogether. In addition, mechanical signals resulting from cell compaction may inhibit YAP independently of LATS kinases (10, 15). When cells are sparse and cell-cell contacts are rare, the Hippo pathway is off, YAP is active, and cells proliferate. Thus, understanding how Hippo pathway activity is tuned in response to cell-cell contact and density within an epithelium could provide crucial insight into organ development, tumorigenesis, and tissue repair.

While much has been learned about upstream molecular and cellular components required for activation of the Hippo core kinase complex, much less is known about molecular and cellular determinants that either turn off the Hippo pathway or limit Hippo pathway activity. The Ras-associated protein Rassf6 negatively regulates Hippo pathway signaling in mammals by antagonizing WW45 binding to Mst2 (16). Salt-inducible kinases inhibit Hippo signaling in Drosophila by phosphorylating Sav and thereby inhibiting Hpo/Wts association (17). The phosphatase PTPN14 promotes nuclear-to-cytoplasmic trafficking of YAP, but the phosphatase activity may not be necessary for it to inhibit Hippo signaling (18, 19). Finally, members of the AJUBA family of LIM domain-containing proteins inhibit Hippo signaling at the level of the core kinases (20). For all these negative regulators, the precise environmental or developmental signal or context that influences their activity, and how, is not fully understood.

There are three mammalian members of the AJUBA LIM protein family—AJUBA, LIMD1, and WTIP—and one Drosophila ortholog, encoded by *dJub. dJub* is an essential gene for Drosophila embryo development, for reasons not fully understood (20, 21). Conditional depletion of *dJub* in developing organs, however, results in a decrease in organ size through a genetic interaction with the Hippo pathway (20). Genetic-epistasis experiments and protein-protein interaction studies indicate that the AJUBA LIM proteins inhibit the Hippo pathway at the level of the core kinase complex (20). Phosphorylation of AJUBA LIM proteins by either enhanced green fluorescent protein receptor (EGFR)-stimulated MAPK (22) or JNK (23, 24) promotes binding of AJUBA LIM proteins and *dJub* to LATS and *Wts*. In Drosophila tissues, increases in cytoskeletal tension inhibit Hippo signaling through induction of a dJub-Wts complex (25). We set out to determine the molecular mechanisms and the cell and developmental context by which AJUBA LIM proteins inhibit the Hippo pathway during epithelial cell-cell CIP.

## MATERIALS AND METHODS

### Cell culture and transfections.

MCF10A cells were cultured in Dulbecco's modified Eagle's medium (DMEM)–F-12 (1:1; Gibco) supplemented with 5% heat-inactivated horse serum (Gibco), 100 ng/ml cholera toxin, 10 μg/ml insulin, 20 ng/ml epidermal growth factor (EGF), 500 ng/ml hydrocortisone, and penicillin-streptomycin (Gibco). HEK293T cells were cultured in DMEM (Gibco) supplemented with 10% heat-inactivated fetal bovine serum (FBS), 200 μM l-glutamine (Cellgro), and penicillin-streptomycin.

Lipofectamine RNAiMax (Invitrogen) was used to transfect MCF10A cells with small interfering RNA (siRNA) oligonucleotides according to the manufacturer's instructions. For density experiments, equal numbers of cells were transfected and plated on dishes of different sizes to provide cells at low density (LD) and high density (HD). All experiments were conducted 48 h posttransfection. TransIt LT1 reagent (Mirus) was used to transfect HEK293T cells with the plasmids indicated in Fig. 4A to 6 and 9 according to the manufacturer's instructions.

### Cell proliferation and apoptosis analysis.

Cell proliferation was measured using BrdU (5-bromo-2′-deoxyuridine) Labeling and Detection kit 1 (Roche). MCF10A cells were plated to achieve the required density conditions and at analysis time were incubated with BrdU-containing medium for 1 h and then fixed in 15 mM glycine dissolved in absolute ethanol at pH 2.0 for 60 min at −20°C. The fixed cells were incubated with anti-BrdU solution for 30 min at 37°C and with anti-Ig-fluorescein for 10 min at 37°C. Cell apoptosis and death were assessed using an annexin V-EGFP apoptosis detection kit (Abcam; ab14153). The cells were plated at the required densities and incubated with annexin V-EGFP for 5 min after washing with phosphate-buffered saline (PBS). In both cases, the cells were mounted and imaged as described in “Immunostaining” below.

### YAP-TEAD-luciferase reporter experiments.

MCF10 cells stably expressing a GTIIC-luciferase TEAD reporter cassette (7) were generated by cloning the GTIIC-luciferase TEAD reporter cassette into a lentiviral vector that contained a blasticidin resistance gene. Lentivirus was produced and used to infect MCF10A cells. Infections were done in the presence of 10 μg/ml protamine sulfate. Forty-eight hours postinfection, MCF10A cells were subjected to selection with 10 μg/ml blasticidin for 4 days. Stable selectants were pooled and maintained in 2.5 μg/ml blasticidin. The same cells were used for each experimental manipulation. For each experiment, MCF10A cells stably expressing the TEAD-luciferase reporter were plated on black-walled 24-well plates at low and high densities. forty-eight hours later, d-luciferin was added, and luciferase activity was measured. Cell numbers were determined by MTT [3-(4,5-dimethyl-2-thiazolyl)-2,5-diphenyl-2H-tetrazolium bromide] assay, and the luciferase flux was normalized to the cell number.

### Immunoprecipitation and Western blotting.

Cells were lysed in RIPA buffer (50 mM Tris, pH 8.0, 150 mM NaCl, 1% NP-40, 0.5% sodium deoxycholate, 0.1% SDS) supplemented with 200 nM phenylmethylsulfonyl fluoride (PMSF), 2 μg/ml aprotinin/leupeptin, 2 μM pepstatin A, 1 mM Na_3_VO_4_, and 2 mM NaF. The lysates were cleared by centrifugation at 12,000 rpm for 10 min, and the concentrations were determined by Bradford assay (Bio-Rad). Equal amounts of protein were boiled in SDS sample buffer, resolved by SDS 8% or 10%-PAGE, and transferred to polyvinylidene difluoride (PVDF) membranes (Millipore) in transfer buffer (25 mM Tris, 192 mM glycine, 5% methanol). The membranes were blocked with TBST (25 mM Tris, pH 7.4, 150 mM NaCl, 2 mM KCl, 0.5% Tween 20) containing 5% skim milk powder or bovine serum albumin (BSA) and probed overnight with the indicated primary antibodies. Bound antibodies were detected by horseradish peroxidase (HRP)-conjugated secondary antibodies and developed with SuperSignal West Pico or West Femto enhanced chemiluminescence (ECL) (Pierce). Images were collected on a Bio-Rad ChemiDoc XRS+ and subjected to quantification using ImageJ software. Student's *t* test was used to calculate the statistical significance of differences in average intensity.

The following antibodies were used: rabbit anti-YAP (Cell Signaling; 4912; 1:1,000), rabbit anti-phospho-YAP (Ser127) (Cell Signaling; 4911; 1:1,000), rabbit anti-LATS1 (Cell Signaling; 3477; 1:500), rabbit anti-AJUBA (Cell Signaling; 4897; 1:1,000), rabbit anti-LIMD1 (26) (1:1,000), mouse anti-Flag M2 (Sigma; F3165; 1:1,000), mouse antihemagglutinin (anti-HA) (Sigma; H3663; 1:1,000), and mouse anti-Myc 9E10 (Millipore; 05-419; 1:1,000). Rabbit anti-LATS2 (1:1,000), rabbit anti-phospho-LATS2 T1041 (1:250), and rabbit anti-phospho-LATS2 S872 (1:250) were from H. Nojima (Osaka University, Osaka, Japan); rabbit anti-WW45 (1:1,000) was from G. Pfeifer (Beckman Research Institute of the City of Hope, Duarte, CA).

For immunoprecipitations (IP), cells were lysed in IP buffer (10 mM HEPES, pH 7.4, 150 mM NaCl, 10% glycerol, 1% NP-40, or CHAPS {3-[(3-cholamidopropyl)-dimethylammonio]-1-propanesulfonate}) supplemented with 200 nM PMSF, 2 μg/ml aprotinin/leupeptin, 2 μM pepstatin A, 1 mM Na_3_VO_4_, and 2 mM NaF. The lysates were cleared by centrifugation at 12,000 rpm for 10 min, and concentrations were determined by Bradford assay (Bio-Rad). Equal amounts of protein were collected, 10% of which was boiled in SDS sample buffer and saved as input, while the remainder was incubated with the indicated antibodies, with rocking, overnight at 4°C. The next day, protein G-conjugated Sepharose beads (Sigma) were added to each reaction mixture at 5 μl bed volume/200 μg lysate and rocked for 1 h at 4°C. The immunoprecipitates were washed 3 times with 1.5 ml IP buffer, followed by centrifugation at 100 × *g* for 1 min.

### Immunostaining.

Cells plated on glass coverslips were fixed in 4% paraformaldehyde (PFA) dissolved in PBS and permeabilized with 0.2% Triton X-100 in PBS. All the cells were blocked in 5% normal goat serum (NGS) for 1 h at room temperature and incubated with the indicated primary antibodies, diluted in PBS plus 1% NGS, overnight at 4°C. Coverslips were washed 3 times with PBS and then incubated with secondary antibody diluted in PBS plus 1% NGS for 1 h at room temperature. The cells were mounted in Vectashield mounting medium containing 4′,6-diamidino-2-phenylindole (DAPI) (Vector Laboratories). Images were collected on an LSM 510 Zeiss confocal microscope using a 40× oil objective. ImageJ was used to process the images.

The following antibodies were used: mouse anti-E-cadherin (BD Biosciences; 610182; 1:500), rabbit anti-LATS1 (Cell Signaling; 3477; 1:500), rabbit anti-LIMD1 (PVDF purified; 1:50), mouse anti-HA (Sigma H3663; 1:500), mouse anti-Myc 9E10 (Millipore; 05-419; 1:500), and rabbit anti-Flag (Sigma; F7425; 1:500). Secondary antibodies were conjugated to Alexa Fluor 488 and 568 (Invitrogen; 1:250).

### Transgenic fly lines.

All crosses and staging were performed at 25°C. The *w1118* line was used as the wild type (wt). The stocks are described in FlyBase (http://flybase.org/). The *Ex697* line was kindly provided by the Bloomington Drosophila Stock Center (B no. 44248). *En-Gal4* and upstream activation sequence (UAS) *GFP* flies were provided by J. Skeath (Washington University School of Medicine, St. Louis, MO). UAS-*HA-hLIMD1* flies were described previously (20). To generate transgenic human LIMD1 (hLIMD1) mutant flies, hLIMD1 mutant cDNA was cloned into pUAST-HA, and the resulting vector was used to generate transgenic lines via standard P element-mediated transformation (Rainbow Transgenics, Inc.). Gene overexpression and RNA interference (RNAi) rescue assays were carried out using the GAL4/UAS system. The GAL4 driver line used was *1096-gal4 engrailed-gal4* UAS-*GFP*. The UAS lines used were UAS-*djubRNAi* (22.5), UAS-*dcr*, UAS-*HA.hLIMD1*, and UAS-*hLIMD1* domain mutants.

### Drosophila adult wing dissection, imaging, and image processing.

Adult flies were stored in 80% ethanol until they were ready for dissection. Only female flies were used for analyses. Wings were removed in 75% glycerol (in PBS) for mounting. Coverslips were sealed with nail polish. The total wing area was measured, and the average and standard deviation were plotted using ImageJ and Microsoft Excel. Student's *t* test was used to calculate the statistical significance of differences in the area of the wing region between various genotypes.

### Drosophila larval wing disc dissection, imaging, and image processing.

Wandering 3rd-instar larval wing discs were dissected in PBS, fixed in 4% paraformaldehyde diluted in PBS for 40 min, washed once for 5 min in PBX (PBS with 0.1% Triton X-100), twice for 20 min in PAXD (PBS with 1% bovine serum albumin, 0.3% Triton X-100, and 0.3% deoxycholate), and once in PAXDG (PAXD with 5% normal goat serum), all on ice. The tissues were incubated overnight in primary antibody diluted in PAXDG at 4°C and washed three times in PBX at room temperature. After >4 h of incubation in secondary antibody diluted in PAXDG at 4°C, they were washed twice in PBX and once in PBS, all at room temperature. The prepared tissues were mounted in Vectashield mounting medium (Vector Laboratories, Burlingame, CA). The antibodies used were rat anti-DE-cadherin (1:20; from the Developmental Studies Hybridoma Bank at the University of Iowa) and rabbit anti-β-galactosidase (1:2,000; ICN/Cappel); the secondary antibodies were conjugated to Alexa Fluor 568 (Invitrogen) and Cy5 (Jackson ImmunoResearch). Immunofluorescence was acquired on a Zeiss LSM 510 confocal microscope. Image J64 (National Institutes of Health, Bethesda, MD) was used to adjust the brightness and contrast of whole images.

### Extracellular matrix (ECM) stiffness experiments.

Polyacrylamide (PA) gels were prepared based on published protocols (27, 28). Solutions with acrylamide/bisacrylamide ratios of 5% to 0.1% and 15% to 1.2% were prepared to make 800-Pa and 120-kPa PA gels, respectively (Sigma-Aldrich). An amount of PA solution adequate to yield a final gel thickness between 100 and 200 μm was sandwiched between a silanized coverslip (3-aminopropyltimethoxysilane; Sigma-Aldrich) and a siliconized glass slide (Sigmacote; Sigma-Aldrich), and the gels were allowed to polymerize using APS and TEMED (Sigma-Aldrich). Coverslips with covalently bonded PA gels were carefully removed from the siliconized glass slides and rinsed in PBS. Fibronectin was covalently linked to the PA gel using the heterobifunctional cross-linker Sulfo-SANPAH (Sigma-Aldrich) and overnight incubation with 50 μg/ml fibronectin (BD Biosciences) solution at 4°C.

### Microfabricated pillar experiments.

Micropatterned glass coverslips with four different sizes of fibronectin-coated pillars in each quadrant of the coverslip were purchased from Cytoo SA. For this experiment, 60,000 immortalized cancer-associated fibroblasts (CAFs) were plated in 6-well dishes containing Cytoo SA micropatterned coverslips. The cells were allowed to attach for 3 h, and then the cell-containing culture medium was removed and replaced with cell-free culture medium. The cells were fixed 7 h after seeding and stained with phalloidin 488 (Invitrogen), DAPI, and anti-YAP (Cell Signaling; 4912; 1:250) according to standard protocols. Imaging was performed on a Nikon Eclipse Ti-E inverted epifluorescence microscope controlled by MetaMorph software (version 7.7.0.0; Molecular Devices). Images were acquired using metal halide lamp illumination (Prior); Semrock Brightline filter sets; a Nikon Plan Fluor 10× air, 20× air, or 40× water immersion objective (numerical apertures [NA], 0.3, 0.45, and 1.25, respectively); and a cooled charge-coupled-device (CCD) camera (CoolSnap HQ2; Photometrics).

### Quantification of YAP subcellular localization.

For automatic quantification of YAP localization, custom Matlab (MathWorks Inc.) software (employing thresholding and morphological closing) was used to identify nuclei and cell boundaries from DAPI and phalloidin immunofluorescence images, respectively. Cytoplasmic regions were defined as the area within cell boundaries that did not overlap nuclei. The nuclear-to-cytoplasmic brightness ratio was defined as the quotient of the mean YAP fluorescence intensity within the nuclear region and the mean YAP fluorescence intensity within the cytoplasmic region.

## RESULTS

### AJUBA LIM proteins influence YAP activity in proliferating, but not growth-arrested, epithelial cells.

**FIG 1:**
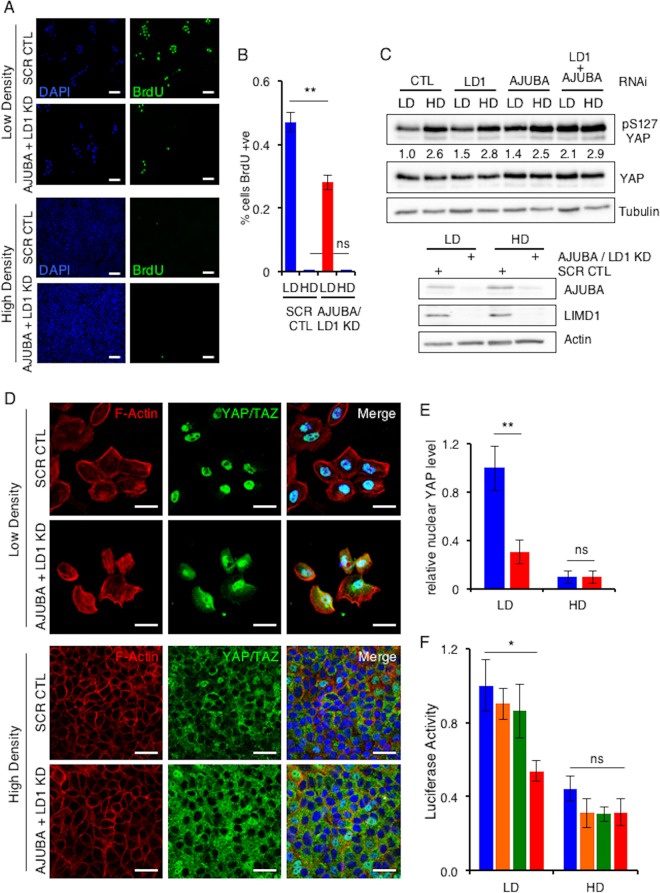
AJUBA LIM proteins regulate YAP activity in proliferating epithelial cells but not growth-arrested epithelial cells. (A) MCF10A cells transfected with control (CTL) scrambled RNAi (SCR) or AJUBA plus LIMD1 (LD1) RNAi were plated at low or high density for 24 h, and BrdU uptake was measured. +ve, positive. (B) Quantification of results in panel A. At least 100 cells were scored under each condition. (C) MCF10A cells were transfected with the indicated RNAi. Control cells were transfected with a scrambled RNAi. After 24 h, the cells were split and cultured at either LD or HD for another 24 h. The cells were lysed, and Western blotting with the indicated antibodies was performed. The pS127YAP/total YAP ratio is shown below each lane. The pYAP/YAP level ratio in control cells at LD was arbitrarily set as 1. (D) The same cells as in panel B were stained with YAP antibody or phalloidin (F-actin), and an immunofluorescence assay was performed. Nuclei were identified with DAPI stain. (E) Quantification of results in panel D. Relative nuclear YAP levels are presented. The amount of nuclear YAP in control LD cells was arbitrarily set as 1. At least 100 cells were scored under each condition. Blue bars, control cells; red bars, AJUBA plus LD1 KD cells. (F) MCF10A cells stably expressing a GTIIC-luciferase TEAD reporter cassette were transfected with the same set of RNAi as in panel C and then plated at LD or HD. The bioluminescence per group was determined, and the results are reported as relative luciferase activity. CTL cells at LD were arbitrarily set to 1. **, *P* < 0.01; *, *P* < 0.05; ns, no significant difference. Each experiment was performed at least 3 times, and a representative example is shown. The data are presented as means ± standard deviations (SD). Scale bars, 100 μm (A) and 50 μm (D). Blue bars, control cells; orange bars, AJUBA KD cells; green bars, LD1 KD cells; red bars, AJUBA plus LD1 KD cells.

To determine how AJUBA LIM proteins limit Hippo pathway activity in mammalian epithelial cells, we studied CIP, a well-described Hippo pathway-regulated event (7, 11). Human MCF10A breast epithelial cells were cultured at various densities: (i) sparse or LD, in which cells are not in contact and proliferate, as determined by BrdU uptake (Fig. 1A); (ii) confluent, in which cell-cell adhesions have formed and proliferation has begun to decrease; and (iii) compacted or HD, where cell proliferation has ceased, as determined by BrdU uptake (Fig. 1A).

**FIG 2:**
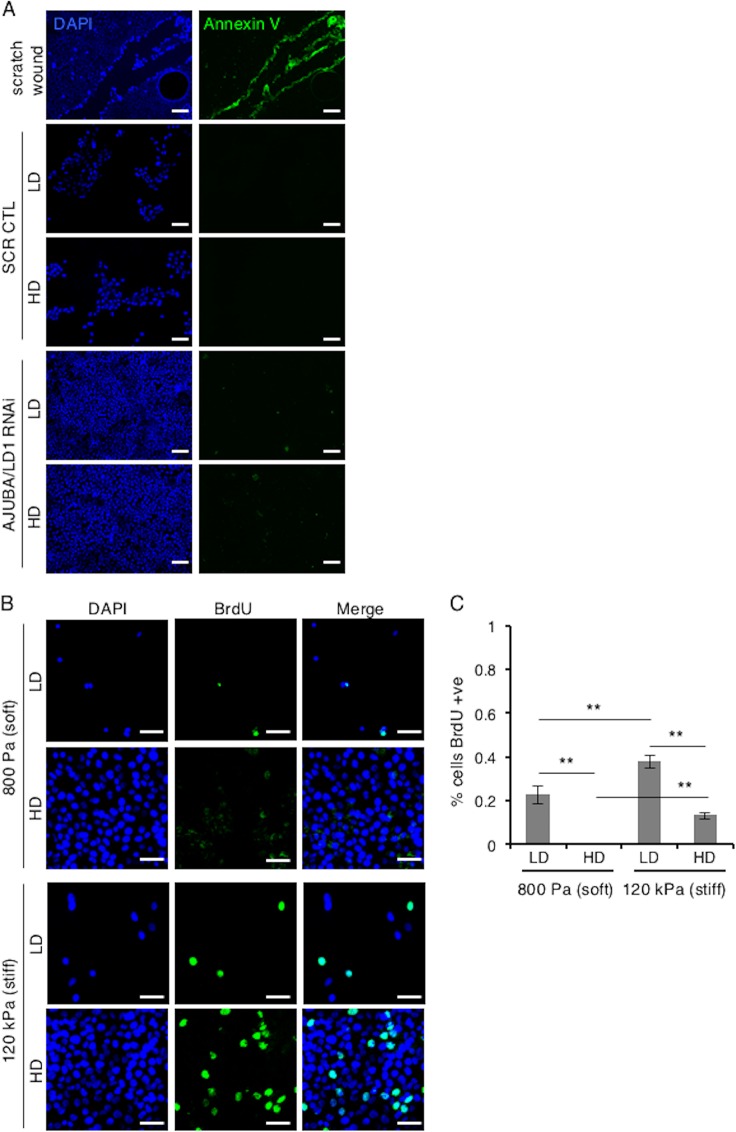
MCF10A cells proliferate in response to exposure to stiff ECM. (A) MCF10A cells transfected with control scrambled RNAi or AJUBA plus LIMD1 RNAi were plated at LD and HD. The extent of apoptosis was determined by annexin V staining. The positive control for apoptosis was a scratch wound assay of MCF10A cells. Note that cells at the edge of the wound underwent apoptosis. (B) MCF10A cells were plated at LD or HD on soft (80- to 120-Pa) or stiff (120-kPa) fibronectin-coated polyacrylamide hydrogels for 24 h. (C) BrdU uptake was then measured and quantified as percent cells BrdU positive. Each experiment was performed at least 3 times, and representative examples are shown. The data are presented as means ± SD. Scale bars, 100 μm (A) and 50 μm (B).

In proliferating LD cells, there was a low level of inhibitory YAP phosphorylation (pS127.YAP) (Fig. 1C) and YAP was predominantly nuclear (Fig. 1D; quantified in Fig. 1E) and transcriptionally active (Fig. 1F). In HD growth-arrested cells, the pS127.YAP level was higher, the nuclear YAP level decreased, and YAP transcriptional activity decreased (Fig. 1C to F). When AJUBA and LIMD1, two of the three mammalian AJUBA LIM proteins, were RNAi depleted individually, there was no significant change in the pS127.YAP level or YAP transcriptional activity in cells at LD or HD (Fig. 1C and F). However, when both AJUBA and LIMD1 were RNAi depleted in LD cells, the level of pS127.YAP increased, nuclear YAP levels decreased, and YAP transcriptional activity decreased (Fig. 1C to F). The pS127.YAP level in AJUBA/LIMD1-depleted LD cells approached that detected in control growth-arrested cells at HD (Fig. 1C). Depletion of both AJUBA and LIMD1 in nonproliferating HD cells did not result in any change in the pS127.YAP level, the YAP nuclear level, or YAP transcriptional activity (Fig. 1C to F). Consistent with the increase in the pS127.YAP level and decreased nuclear YAP in LD cells depleted of AJUBA/LIMD1, cell proliferation decreased, while in HD cells, depletion of AJUBA and LIMD1 did not affect cell proliferation, as determined by BrdU uptake (Fig. 1A; quantified in Fig. 1B). We were unable to deplete all three mammalian AJUBA LIM proteins, as doing so resulted in cell death, in agreement with results in Drosophila, where the single AJUBA LIM gene, *dJub*, is essential for embryonic development (20). There was no increase in cell death in AJUBA/LIMD1-depleted cells, however (Fig. 2A).

These results indicated that in proliferating mammalian epithelial cells the presence of the AJUBA and LIMD1 LIM proteins limited YAP inhibition, thereby sustaining cell proliferation, whereas in growth-arrested epithelium, where YAP was maximally inhibited, the AJUBA and LIMD1 LIM proteins had no further effect upon YAP regulation.

### In mammalian cells, AJUBA LIM proteins do not affect YAP regulation by mechanical signals.

During CIP, cell spreading becomes restricted, as cells are compacted (i.e., high density). This can lead to decreased intracellular tension. Similarly, when cells spread (i.e., low density), intracellular tension increases. In mammalian cells, YAP can be activated by cell-extrinsic and cell-intrinsic mechanical signals (e.g., exposure to a stiff ECM or increased intracellular tension, respectively) (10, 15). Rho GTPase and actin turnover are critical for this response, but whether the Hippo core kinase cascade and LATS are required is not clear (10, 24, 29). Furthermore, during Drosophila wing development, *dJub* was recently shown to be genetically required for intracellular-tension-mediated regulation of Yki (YAP) (25). It was proposed in that study that dJub recruited Wts to α-catenin-dependent junctions in a tension-dependent manner. These observations led us to ask whether, in mammalian epithelial cells, AJUBA LIM proteins affected YAP regulation in response to mechanical signals, which can be present during CIP.

**FIG 3:**
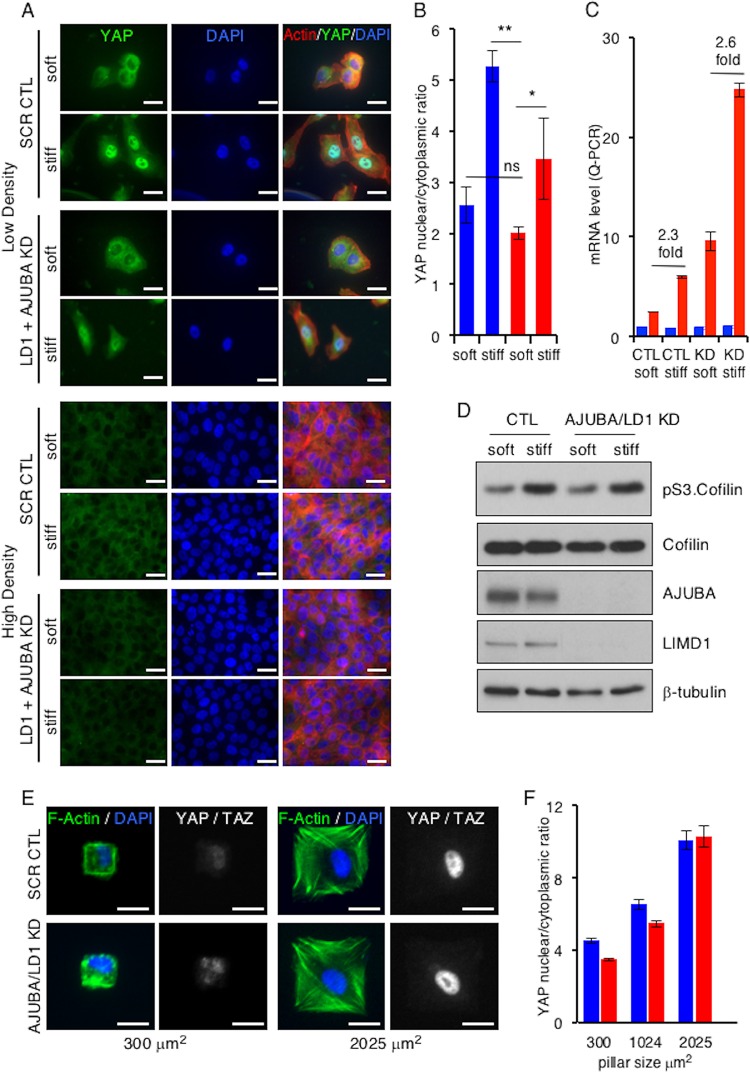
In mammalian cells, AJUBA LIM proteins do not influence mechanical signal regulation of YAP. (A) MCF10A cells were transfected with CTL (scrambled) or AJUBA plus LIMD1 (LD1) RNAi and then split and cultured at either LD or HD on soft (80- to 120-Pa) or stiff (120-kPa) fibronectin-coated polyacrylamide hydrogels for 24 h. Immunofluorescence assays with the indicated antibodies or stains were then performed. (B) Quantification of YAP nuclear/cytoplasmic immunofluorescence from cells in panel A. KD refers to AJUBA and LIMD1 RNAi-depleted cells. At least 50 cells in multiple fields were scored. Blue bars, control cells; red bars, AJUBA plus LD1 KD cells. (C) Quantitative PCR (Q-PCR) for YAP and the CTGF gene (a YAP-regulated gene) in cells from panel A at intermediate density. KD refers to AJUBA and LIMD1 RNAi-depleted cells. Blue bars, YAP mRNA levels; red bars, CTGF mRNA levels. (D) Western blot of cell lysates from cells in panel A with the indicated antibodies. (E) Representative images of CAFs plated on micropatterned coverslips containing fibronectin-coated pillars of different sizes and stained for DAPI, actin, and YAP-TAZ. (F) Quantification of YAP-TAZ nuclear/cytoplasmic ratios from cells in panel D. All the quantified experiments were performed 2 or 3 times, and a representative example is shown. Blue bars, control cells; red bars, AJUBA plus LD1 KD cells. **, *P* < 0.01; *, *P* < 0.05; ns, no significant difference. The data are presented as means ± SD. Scale bars, 25 μm (A) and 20 μm (D).

To determine this, we undertook two approaches. First, we plated MCF10A cells with or without AJUBA and LIMD1 at low and high densities on fibronectin-coated soft and stiff polyacrylamide hydrogels and determined the nuclear/cytoplasmic distributions of YAP. We confirmed that exposure of cells at LD or HD to a stiff ECM increased proliferation, as measured by BrdU incorporation (Fig. 2B; quantified in Fig. 2C). In LD cells on soft matrices, YAP was predominantly cytoplasmic; however, when exposed to a stiff ECM, the nuclear YAP level and transcriptional activity (e.g., connective tissue growth factor [CTGF] gene transcription) increased, as expected (Fig. 3A; quantified in Fig. 3B and C) (10). In LD cells RNAi depleted of AJUBA and LIMD1, the increase in the nuclear YAP level and transcriptional activity in response to shifting from soft to stiff matrices still occurred to the same extent as in control cells (1.9-fold versus 2.1-fold for the nuclear protein level and 2.3-fold versus 2.6-fold for CTGF transcription) (Fig. 3A; quantified in Fig. 3B and C). In cells at HD, there was little nuclear YAP detected, regardless of whether cells were plated on soft or stiff matrices, and depletion of AJUBA and LIMD1 had no effect upon the subcellular distribution of YAP on either soft or stiff matrices (Fig. 3A).

In control experiments, depletion of AJUBA and LIMD1 did not affect the increase in intracellular tension that follows exposure to a stiff environment, as determined by the pS3-cofilin level (Fig. 3D) and F-actin polarization (Fig. 3A). Phospho-S3.cofilin is a downstream target of LIMK (30) that is activated by increased Rho-ROCK in cells exposed to a stiff ECM.

In the second approach, cells were plated on microfabricated pillars or islands of increasing area. On small pillars (300 μm^2^), cells did not spread and intracellular tensions were low, while on larger islands (2,025 μm^2^), cells spread and intracellular tension increased. In cells on small pillars, the nuclear/cytoplasmic ratio of YAP was low, and it increased in cells spread on large pillars, as expected (Fig. 3E; quantified in Fig. 3F) (10). Depletion of AJUBA and LIMD1 did not affect the YAP nuclear/cytoplasmic distribution in response to increasing intracellular tension (Fig. 3E; quantified in Fig. 3F).

These results indicated that in mammalian MCF10A breast cells, AJUBA LIM proteins did not influence YAP activity following exposure to two different mechanical signals: a cell-extrinsic signal (stiff ECM) and a cell-intrinsic signal (intracellular tension).

### AJUBA LIM proteins inhibit activation of LATS by the core Hippo kinase complex in proliferating, but not growth-arrested, cells.

**FIG 4:**
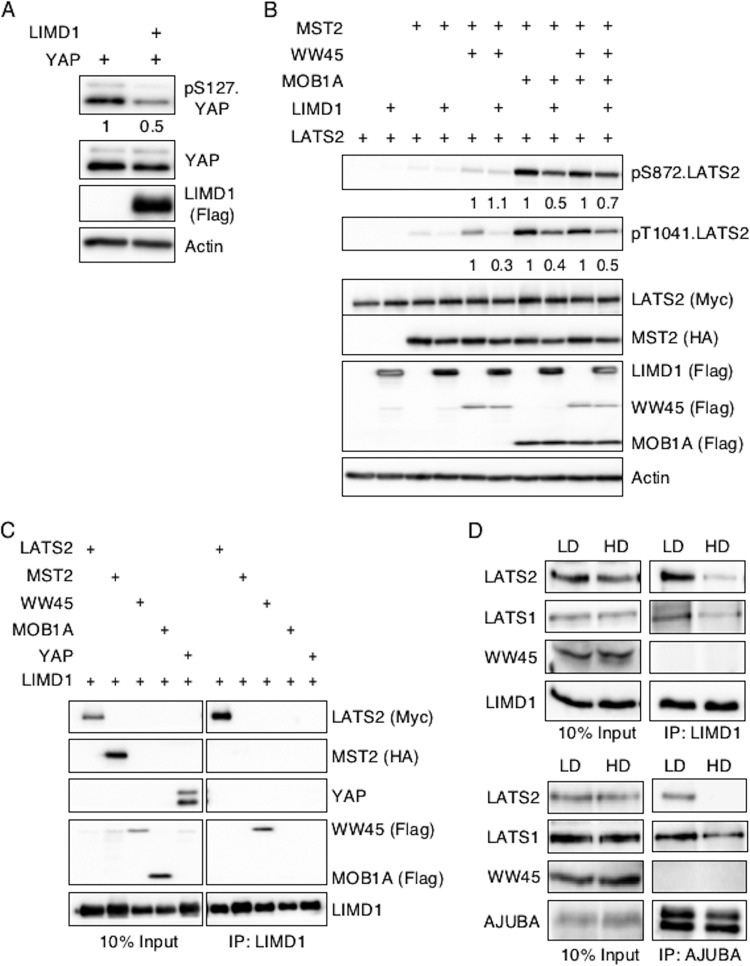
AJUBA LIM proteins inhibit activation of LATS by the core Hippo kinase complex and associate with LATS in proliferating cells but not growth-arrested cells in contact. (A) HEK293T cells were transfected with YAP with or without LIMD1, and the cell lysates were Western blotted with the indicated antibodies. The amount of pS127.YAP detected was controlled for the level of total YAP. The pS127YAP/total YAP ratio is shown below each lane. The amount present in cells not transfected with LIMD1 was arbitrarily set as 1. (B) HEK293T cells were transfected with different combinations of epitope-tagged plasmids expressing components of the Hippo core kinase complex, as indicated, with or without LIMD1. The cell lysates were Western blotted with the indicated antibodies. The amount of active LATS (pS872 and pT1041) in the absence of LIMD1 (equal to 1 for each set) versus the presence of LIMD1, controlled for total LATS2 protein present, was quantified. The relative amount of pS872.LATS2 or pT1041.LATS2 detected in each pair is shown below the top two panels. The amount of phospho-LATS2 species detected in cells not transfected with LIMD1 was arbitrarily set as 1 for each set. All phospho-LATS2 species amounts were normalized to total LATS2 level. (C) HEK293T cells were transfected with LIMD1 and individual components of the Hippo core kinase complex or YAP, as indicated. LIMD1 was immunoprecipitated from the cell lysates, and the bound products were Western blotted with the indicated antibodies. The left column is 10% of the amount of cell lysate used in the IP as an input control. (D) MCF10A cells grown at LD or HD were lysed, and either AJUBA or LIMD1 was immunoprecipitated. The bound products were Western blotted with the indicated antibodies. The left column shows input cell lysate controls (10% of the amount used for the IP).

To determine if the AJUBA LIM proteins inhibited Hippo pathway regulation of YAP in mammalian cells, and how, we reconstituted Hippo signaling in proliferating transformed HEK293T epithelial cells. Since LIMD1 is the mammalian AJUBA LIM protein most closely related to dJub, we used LIMD1 as the AJUBA LIM protein for all subsequent experiments. When cells were transfected with YAP alone or YAP and LIMD1, the presence of LIMD1 resulted in a 50% reduction in the pS127.YAP level (Fig. 4A), consistent with its role in regulating YAP in proliferating MCF10A cells (Fig. 1).

The Hippo pathway consists of a core multiprotein kinase complex. MST kinases phosphorylate and activate LATS kinases, which in turn phosphorylate YAP at S127 and other sites. The adapter protein WW45 modulates MST kinase activity, while MOB proteins influence LATS kinase activity. Phosphorylation of S127 in YAP leads to its nuclear exclusion and, thus, inhibition of transcriptional activity (i.e., the Hippo pathway inhibits YAP activity). Epitope-tagged MST2, WW45, LATS2, and MOB1A were cotransfected into HEK293T cells in different combinations, and LATS2 activity in total cell lysates was determined by Western blotting with antibodies specific for active LATS2: pT1041.LATS2 and pS872.LATS2 (16, 31). Expression of MST2 and MOB1A was sufficient to induce maximal LATS2 activation (Fig. 4B). The addition of WW45 did not further enhance LATS2 activity (Fig. 4B). Neither MOB1A nor MST2 alone activated LATS2 (Fig. 4B). Expression of LIMD1 under all conditions that activated LATS2 decreased the amount of total cellular active LATS2 (Fig. 4B). There also appeared to be a subtle decrease in the molecular size of MST2 in the presence of LIMD1, suggesting that LIMD1 may also affect MST2 phosphorylation (and possibly activity) when associated with the core Hippo kinase complex (Fig. 4B).

In sum, we were able to reconstitute Hippo pathway regulation of LATS activity in HEK293T cells, and in this reconstituted system, the AJUBA LIM protein LIMD1 inhibited LATS activation by the Hippo core kinase complex, and thus, likely YAP inactivation.

### AJUBA LIM proteins preferentially associate with LATS kinases in proliferating, but not growth-arrested, cells.

Genetic-epistasis experiments in Drosophila revealed that *dJub* (the single ortholog of mammalian AJUBA LIM protein family genes) inhibits Hippo pathway signaling at the level of the core kinase complex upstream of *Yki* (the mammalian YAP/TAZ gene) (20). To determine how AJUBA LIM proteins inhibited the activation of LATS, we first asked which components of the Hippo kinase complex the AJUBA LIM proteins interacted with. When LIMD1 was expressed with LATS2, MST2, WW45, MOB1A, or YAP individually, LIMD1 was found to associate with LATS2 and WW45 but not MST2, MOB1A, or YAP in coimmunoprecipitation experiments (Fig. 4C). To confirm these interactions in cells expressing endogenous levels of Hippo pathway components and AJUBA LIM proteins (i.e., no overexpression), we turned to MCF10A cells. AJUBA LIM protein inhibited YAP phosphorylation only in proliferating cells (Fig. 1), and if an association between AJUBA LIM proteins and LATS is critical for their capacity to inhibit LATS activation, then one would predict that AJUBA LIM protein would preferentially associate with LATS in proliferating cells, as opposed to growth-arrested cells. Significantly more LATS2 was indeed coimmunoprecipitated with LIMD1 or AJUBA from proliferating LD MCF10A cells than from growth-arrested HD cells (Fig. 4D). The amount of LATS1 that coimmunoprecipitated with LIMD1 or AJUBA in proliferating LD cells was also greater than in growth-arrested HD cells, but the difference was not as great as that seen with LATS2 (Fig. 4D). Endogenous WW45 was not detected in LIMD1 immunoprecipitates regardless of whether cells were growing under low- or high-density conditions (Fig. 4D).

In sum, these results indicated that AJUBA LIM proteins preferentially associated with LATS1/LATS2 in proliferating cells, not in growth-arrested cells.

### AJUBA LIM proteins sequester LATS2 in a Hippo core kinase complex in the cytosol.

All four components of the Hippo core kinase complex form a physical complex in cells, and formation of this complex is thought to be important for activation of LATS (32). Since AJUBA LIM proteins are molecular adaptors, we asked whether they might inhibit LATS activation by altering the associations between various “upstream” components within the Hippo core kinase complex.

**FIG 5:**
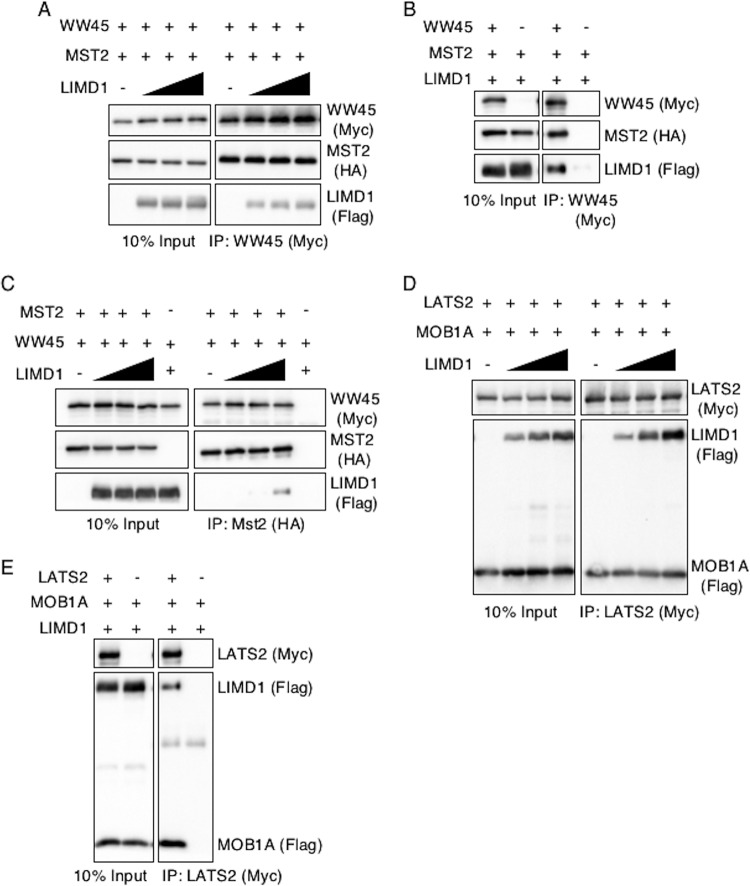
AJUBA LIM proteins do not disrupt MST2-WW45 and MOB1A-LATS2 interactions in cells. (A to C) HEK293T cells were transfected with epitope-tagged plasmids expressing WW45 or MST2 in the absence or in the presence of increasing amounts of LIMD1. The cells were lysed, and either WW45 or MST2 was immunoprecipitated. The bound products were Western blotted with the indicated antibodies. The left columns are input controls (10% of the amount of cell extract immunoprecipitated). (D and E) HEK293T cells were transfected with epitope-tagged plasmids expressing LATS2 or MOB1A in the absence or in the presence of increasing amounts of LIMD1. The cells were lysed, and LATS2 was immunoprecipitated. The bound products were Western blotted with the indicated antibodies. The left columns are input controls (10% of the amount of cell extract immunoprecipitated).

MST phosphorylates and activates LATS, and the interaction between WW45 and MST2 stimulates MST2 activity. The presence of increasing amounts of transfected LIMD1 did not affect the amount of WW45 that associated with MST2 regardless of whether WW45 or MST was immunoprecipitated (Fig. 5A to C). The interaction of MOB1A with LATS stimulates LATS activity. The presence of increasing amounts of transfected LIMD1 did not affect the association of MOB1A with LATS2 (Fig. 5D and E).

**FIG 6:**
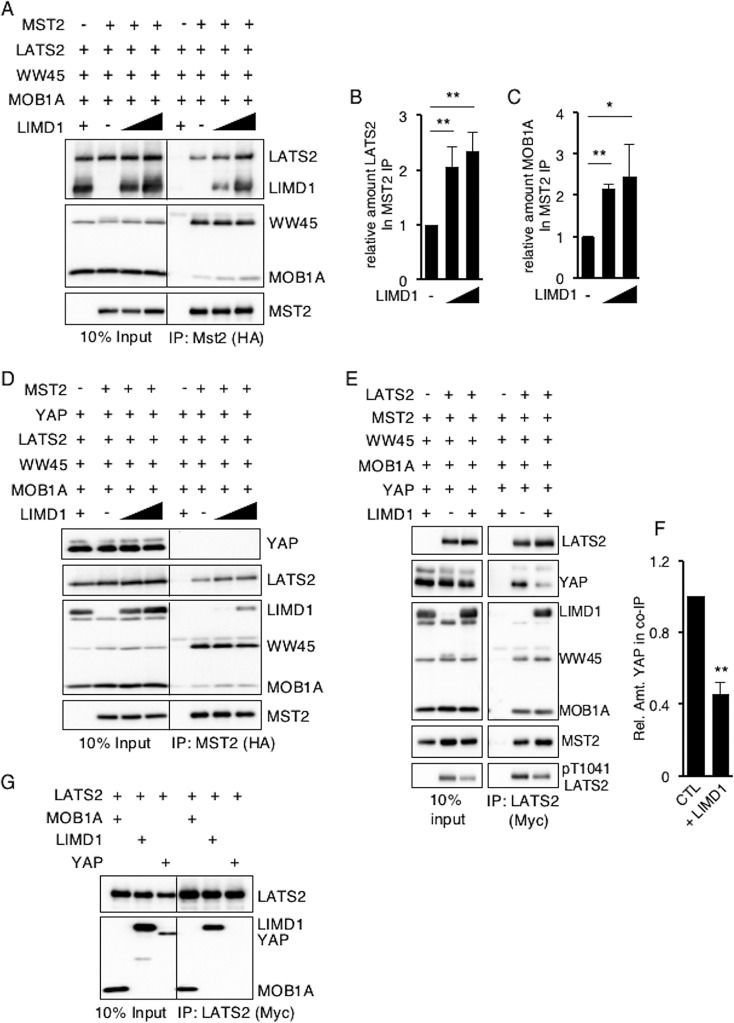
In proliferating cells, AJUBA LIM proteins sequester LATS in a Hippo core kinase complex that does not contain YAP. (A) HEK293T cells were transfected with epitope-tagged Hippo core kinase complex plasmids, with or without LIMD1, as indicated. The cells were lysed, MST2 was immunoprecipitated, and the bound products were Western blotted with the indicated antibodies. The left column is the input controls (10% of the amount of cell lysate used for IP). (B and C) Quantification of the relative amounts of LATS2 (B) and MOB1A (C) in MST2 IP in the presence of increasing amounts of LIMD1. The value in cells not transfected with LIMD1 was arbitrarily set as 1. (D) The same experiment as in panel A, but all the cells were also transfected with a YAP-expressing plasmid. (E) HEK293T cells were transfected with epitope-tagged Hippo core kinase complex plasmids with or without LIMD1, as indicated. The cells were lysed, LATS2 was immunoprecipitated, and the bound products were Western blotted with the indicated antibodies. The left column is the input controls (10% of the amount of cell lysate used for IP). (F) Quantification of the relative amounts of YAP in LATS2 IP in the absence (CTL) or presence of LIMD1. The value in cells not transfected with LIMD1 (CTL) was arbitrarily set as 1. (G) All HEK293T cells were transfected with epitope-tagged LATS and then MOB1A, LIMD1, or YAP individually. LATS2 was immunoprecipitated from the cell extracts, and the bound products were Western blotted with the indicated antibodies. **, *P* < 0.01; *, *P* < 0.05. All the quantified experiments were performed at least 3 times, and a representative example is shown. The data are presented as means and SD.

When all four components of the Hippo core kinase complex were cotransfected and MST2 immunoprecipitated, WW45, LATS2, and MOB1A all coimmunoprecipitated with MST2 (Fig. 6A). In the presence of increasing amounts of LIMD1, the amounts of LATS2 and MOB1A present in MST2 immunoprecipitates increased (Fig. 6A; quantified in Fig. 6B and C, respectively). LIMD1 was also present in the MST2 immunoprecipitate (Fig. 6A). YAP was not detected in MST2 immunoprecipitates, regardless of the absence or presence of LIMD1 (Fig. 6D). When the same experiment was repeated but the core kinase complex immunoprecipitated with LATS2 instead of MST2, YAP was present, and in the presence of LIMD1, the amount of YAP in the immunoprecipitate decreased by ∼60% and the amount of active LATS2 (e.g., pT1041) was decreased, despite increased LATS2 (Fig. 6E; quantified in Fig. 6F). YAP did not associate with LATS2 in coimmunoprecipitation experiments when just the two were transfected in the absence of other Hippo core kinase complex components, however (Fig. 6G).

These results suggested that in proliferating cells, AJUBA LIM proteins inhibited Hippo pathway-mediated LATS2 inactivation of YAP (i.e., phosphorylation of YAP) by sequestering LATS kinase and MOB1A. Since total cellular active LATS2 was decreased in the presence of LIMD1 (Fig. 4B), this implied that LATS kinase sequestered in an AJUBA LIM protein-Hippo core kinase complex was inhibited from upstream activation.

### AJUBA LIM proteins do not inhibit Hippo-mediated activation of LATS at the plasma membrane.

**FIG 7:**
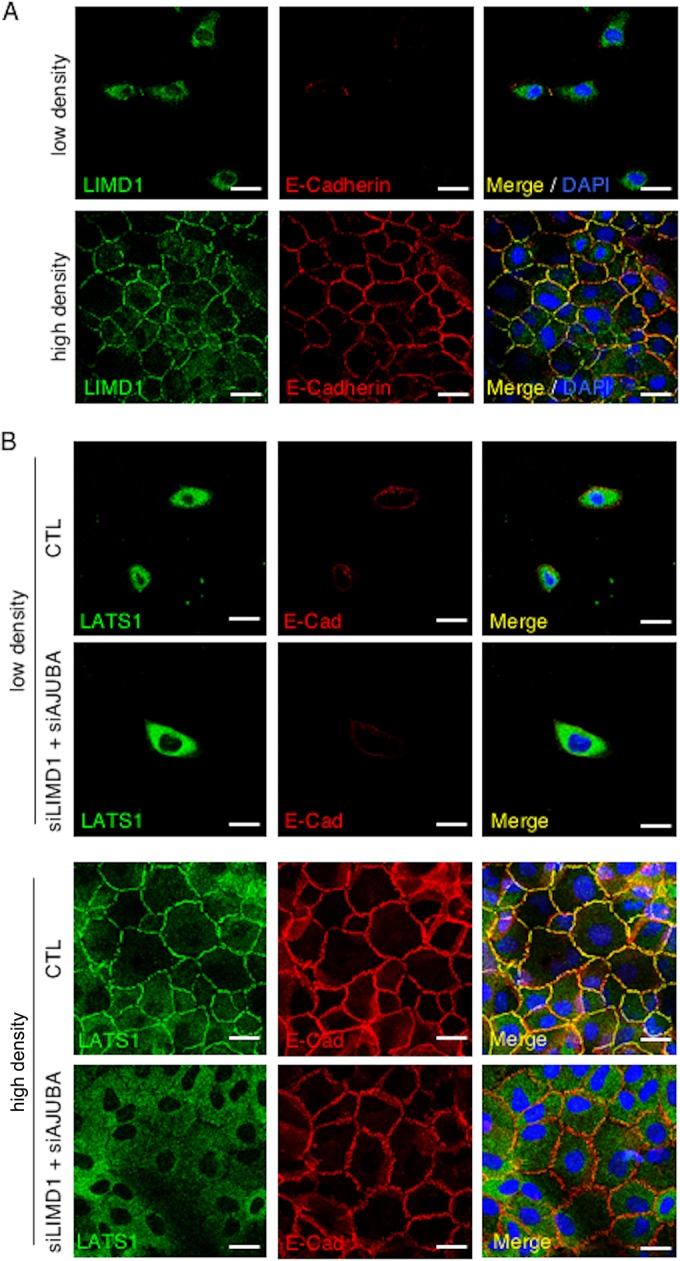
Cellular distribution of LIMD1 and Hippo core kinase complex components in cells. (A) MCF10A cells were cultured at low and high density, and then the cells were fixed and stained with antibodies to LIMD1 and E-cadherin. Immunofluorescence assays were performed. Nuclei were visualized with DAPI stain. (B) MCF10A cells transduced with scrambled RNAi (CTL) or AJUBA and LIMD1 RNAi were plated at LD or HD, and the cells were fixed and stained with LATS1 and E-cadherin antibodies. Immunofluorescence assays were performed. Nuclei were visualized with DAPI stain. Scale bars, 50 μm.

A number of studies have indicated that LATS kinase activation by the Hippo pathway may be spatially restricted. It has been argued that cytosolic LATS is inactive and recruitment to the plasma membrane by NF2 leads to its activation by MST kinases at the plasma membrane (33). Hippo core kinase components also interact with cell surface membrane-associated proteins, including at sites of cell-cell adhesion (34, 35). In LD proliferating MCF10A cells, AJUBA LIM proteins (LIMD1) were cytosolic (Fig. 7A) (36); however, as epithelial cells form adherent, confluent sheets, AJUBA LIM proteins (LIMD1) were recruited to the plasma membrane through an association with α-catenin present at adherens junctions (AJ) (Fig. 7A) (37). However, AJUBA LIM proteins associated with LATS and inhibited LATS activation only in proliferating MCF10A cells (Fig. 1B and 4B), where AJUBA LIM proteins and LATS kinases are predominantly cytosolic (Fig. 7A and B). In growth-arrested HD MCF10A cells, the AJUBA LIM protein-LATS association was much reduced, and AJUBA LIM proteins did not inhibit YAP regulation, even though LIMD1 was present at sites of cell-cell adhesion.

We asked whether the presence of AJUBA LIM proteins affected the subcellular localization of LATS kinases. In LD proliferating cells, both endogenous LIMD1 and LATS1 are cytosolic, and depletion of AJUBA and LIMD1 did not affect LATS1 subcellular distribution (Fig. 7A and B). However, in HD growth-arrested cells, depletion of AJUBA and LIMD1 attenuated the plasma membrane localization of endogenous LATS1 (Fig. 7B). Despite this change in LATS1 subcellular localization, depletion of AJUBA LIM proteins did not affect YAP activation in contacted cells undergoing CIP (Fig. 1).

**FIG 8:**
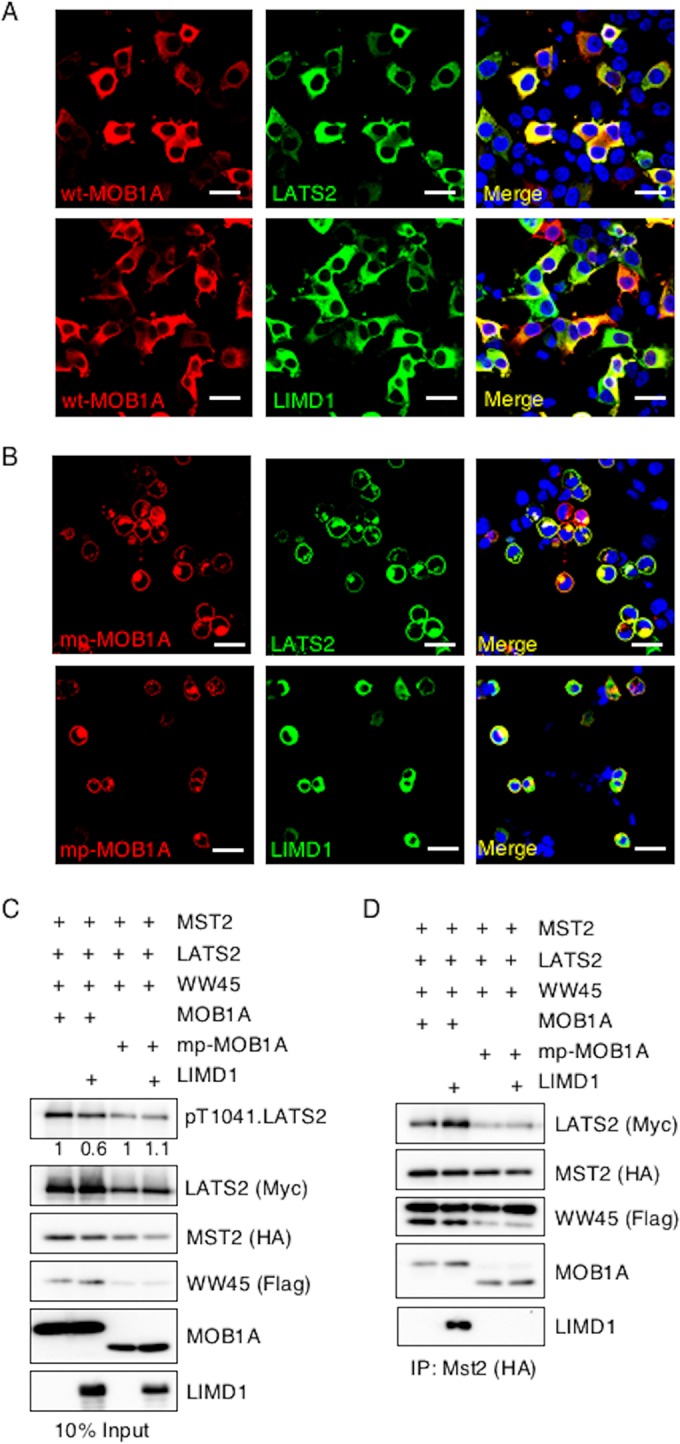
AJUBA LIM proteins do not inhibit Hippo activation of LATS at the plasma membrane. (A and B) HEK293T cells were transfected with MST2, LATS2, LIMD1, and either wt MOB1A (A) or mp-MOB1A (membrane-targeted MOB1A) (B). MOB1A, LATS2, and LIMD1 immunofluorescence assays were performed, as indicated. Nuclei were visualized with DAPI stain. Scale bars, 50 μm. (C and D) HEK293T cells were transfected with MST2, LATS2, or WW45, with or without LIMD1 and with either MOB1A or mp-MOB1A, as indicated. (C) The cells were lysed and Western blotted with the indicated antibodies (input control, 10% of the cell extracts used for IP). (D) MST2 was immunoprecipitated, and the bound products were Western blotted with the indicated antibodies. The amount of pT1041.LATS2 detected is shown below each lane in the top panel. The amount of pT1041.LATS2 present in cells not transfected with LIMD1 was arbitrarily set as 1. All pT1041.LATS2 levels were normalized to total LATS2.

If the ability of LIMD1 to physically interact with LATS2 and the Hippo core kinase complex is critical for its capacity to inhibit activation of LATS, then in confluent epithelial sheets, LIMD1 may not inhibit because it can no longer associate with the Hippo core kinase complex at the plasma membrane. To test this possibility, we activated the Hippo pathway at the plasma membrane by expressing a plasma membrane-targeted isoform of MOB1A, mp-MOB1A. The mp-MOB1A mutant constitutively activates LATS2 at the plasma membrane (38). We determined and contrasted the extent of Hippo core kinase complex-LIMD1 association in cells expressing plasma membrane-targeted constitutively active mp-MOB1A and cells containing wt MOB1A. In HEK293T cells expressing wt MOB1A, MOB1A, LATS2, and LIMD1 all localized to the cytosol (Fig. 8A), whereas in cells expressing mp-MOB1A, mp-MOB1A and LATS2 localized to the plasma membrane but LIMD1 remained largely cytosolic (Fig. 8B).

In cells expressing wt MOB1A, the presence of LIMD1 inhibited total cellular LATS2 activity (Fig. 4B). However, in cells expressing mp-MOB1A, LIMD1 did not inhibit LATS2 activity (Fig. 8C). Of note, in mp-MOB1A-transfected cells uniformly fewer Hippo core kinase complex components were present following transfection than in cells transfected with wt MOB1A cells (Fig. 8C). Regardless, when MST2 was immunoprecipitated from both sets of cells, LIMD1 was detected only in MST2 immunoprecipitates from cells expressing wt MOB1A, not membrane-targeted mp-MOB1A (Fig. 8D). Furthermore, only in the presence of wt MOB1A, not mp-MOB1A, did the presence of LIMD1 increase the amount of LATS2 in the MST2 immunoprecipitate (Fig. 8D).

In summary, when Hippo activation of LATS was generated by plasma membrane targeting of the Hippo core kinase component MOB1A (mp-MOB1A), LIMD1 was not recruited to the plasma membrane, did not associate with LATS or the Hippo core kinase complex, and did not inhibit LATS activation. These results indicated that the capacity of AJUBA LIM proteins to inhibit Hippo pathway activation of LATS kinases is directly correlated with the ability of AJUBA LIM proteins to interact with LATS and the Hippo core kinase complex. Thus, AJUBA LIM proteins do not inhibit Hippo-mediated activation of LATS and YAP regulation at the plasma membrane because at that subcellular location they do not interact with the Hippo core kinase complex.

### Sequestration of LATS2 in a Hippo core kinase complex by LIMD1 correlates with LIMD1's capacity to limit Hippo activity during Drosophila wing development.

**FIG 9:**
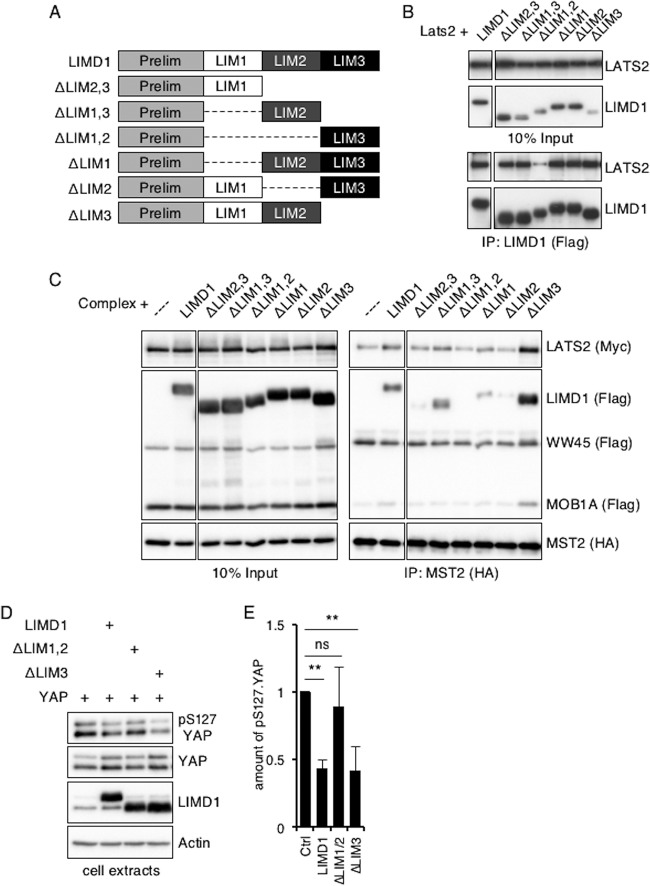
Mapping the LIM region(s) of LIMD1 that mediates association with LATS and the Hippo core kinase complex in cells. (A) Diagram of LIM domain mutants of human LIMD1. (B) HEK293T cells were transfected with LATS2 and the indicated hLIMD1 mutants. LIMD1 was immunoprecipitated, and the bound products were Western blotted with the indicated antibodies. The left column is an input control. (C) HEK293T cells were transfected with epitope-tagged Hippo core kinase complex components and the indicated hLIMD1 mutants. MST2 was immunoprecipitated, and the bound products were Western blotted with the indicated antibodies. The left column is an input control. (D) HEK293T cells were transfected with the indicated plasmids. The cells were lysed, and cell extracts were Western blotted with the indicated antibodies. (E) Quantification of the relative amounts of pS127.YAP, normalized to total YAP protein, in the various lanes of panel D. The amount of pS127.YAP present in control cells (transfected with only YAP) was arbitrarily set as 1. **, *P* < 0.01; ns, no significant difference. The quantified experiments were performed at least 3 times, and a representative example is shown. The data are presented as means and SD.

The C-terminal region of the AJUBA LIM proteins contains three tandem LIM domains, and the region directs its association with LATS2 (39). Since any individual LIM domain is a protein-protein interacting domain (40), we asked which LIM domain, or combination of LIM domains, was required for the association with LATS2 alone and with the complete Hippo core kinase complex. Using a panel of LIM domain deletion mutants of human LIMD1 (Fig. 9A), both the LIM1 and LIM2 domains were found to be required for the efficient association of LIMD1 with LATS2 (Fig. 9B). Deletion of either alone was not sufficient to disrupt the LATS2-LIMD1 association (Fig. 9B). Both the LIM1 and LIM2 domains of LIMD1 were also required for the association of LIMD1 with the MST2-immunoprecipitated Hippo core kinase complex (Fig. 9C), although in this setting, loss of either individual domain decreased the amount of LIMD1 mutant protein that associated with the Hippo core kinase complex (Fig. 9C). Interestingly, removal of the LIM3 domain increased the amounts of LIMD1, LATS2, and MOB1A that associated with the MST2-immunoprecipitated complex (Fig. 9C), suggesting that the LIM3 domain may mask access to the LIM1 and LIM2 domains in cells. The presence of both LIM1 and LIM2 domains was required for LIMD1 to efficiently inhibit S127 phosphorylation of YAP (Fig. 9D; quantified in Fig. 9E).

**FIG 10:**
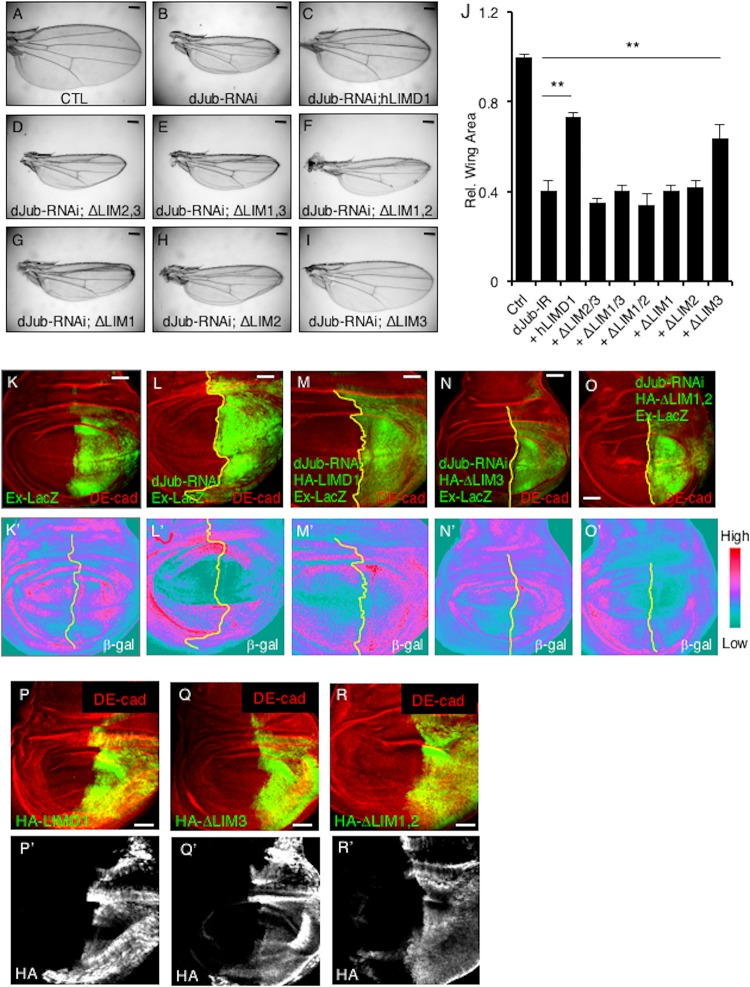
Sequestration of LATS2 in a core Hippo kinase complex by hLIMD1 mutants correlates with the capacity to limit Hippo activity in Drosophila wing imaginal discs. (A to I) Drosophila wings from transgenic flies expressing *dJub*-RNAi and the indicated human LIMD1 mutant. (J) Quantification of the relative wing areas from various mutants. The wing area in wt control flies was set as 1. **, *P* < 0.01. For each genotype, at least 20 wings were scored. All the flies were the same sex and age. The data are presented as means and SD. (K to O and K′ to O′) Confocal immunofluorescent localization of DE-cadherin and beta-galactosidase in 3rd-instar larval wing discs expressing *dJub*-RNAi and the indicated hLIMD1 transgene via En-Gal4 or UAS-GFP in the background of Ex697 (Ex-LacZ). (K) Control Ex-LacZ-expressing wing imaginal disc demonstrating the basal level of LacZ staining. (P to R) Confocal immunofluorescent localization of DE-cadherin and the indicated HA-tagged hLIMD1 transgenes in 3rd-instar larval wing discs via En-Gal4 or UAS-GFP. Scale bars, 200 μm (A to I) and 50 μm (K to R).

We then asked whether these mapping studies correlated with the ability of LIMD1 to limit Hippo activity during Drosophila wing development. Depletion of *dJub* results in small adult wings due to its ability to regulate Hippo pathway activity (20) (Fig. 10B; quantified in Fig. 10J). This phenotype can be rescued by overexpression of human LIMD1 (Fig. 10C; quantified in Fig. 10J) (20). We then tested the various LIM domain deletion mutants of hLIMD1 for the ability to rescue the *dJub*-RNAi small-wing phenotype. Only those that associated with the Hippo core kinase complex and LATS2 (e.g., full-length LIMD1 or lacking LIM3) were able to rescue the small-wing phenotype (Fig. 10C and I; quantified in Fig. 10J). Transgenic hLIMD1 mutants that did not associate with the core Hippo kinase complex (e.g., lacking LIM1, LIM2, or both) did not rescue the small-wing phenotype (Fig. 10D to H; quantified in Fig. 10J).

Deletion of *dJub* resulted in increased Hippo signaling in the wing imaginal disc (i.e., decreased Hippo inhibition), as evidenced by decreased expression of the Yki (YAP) Expanded target gene (*Ex-LacZ*) (Fig. 10K, K′, L, and L′). Expression of wt hLIMD1 and the ΔLIM3 mutant rescued this defect (Fig. 10M and M′ [wt hLIMD1] and N, and N′ [ΔLIM3 mutant]), while ΔLIM1,2 did not (Fig. 10O and O′). Each rescue transgene was expressed, as determined by HA immunofluorescence of wing imaginal discs (Fig. 10P to R).

In summary, the ability of LIMD1 to associate with LATS and the Hippo core kinase complex directly correlated with the ability of hLIMD1 transgenes to rescue the *dJub* small-wing phenotype and inhibit Hippo signaling *in vivo* in the developing Drosophila wing.

## DISCUSSION

Our results indicate that the AJUBA LIM proteins limit Hippo pathway-mediated YAP inactivation in proliferating cells. In growth-arrested cells, CIP-mediated Hippo activation is not inhibited by the presence of AJUBA LIM proteins. Our data suggest a model whereby AJUBA LIM proteins inhibit Hippo core kinase complex activation of YAP in proliferating cells by sequestering the Hippo core kinase complex, including LATS kinase, in the cytosol and inhibiting activation of LATS kinase. At the plasma membrane, where LATS kinases are thought to be activated by the Hippo core kinase complex, AJUBA LIM proteins do not associate with the Hippo core kinase complex or LATS kinases and do not inhibit Hippo pathway-mediated YAP regulation. If a primary function of AJUBA LIM proteins is to limit Hippo pathway inactivation of YAP/TAZ in proliferating cells, then this could explain why *dJub* is required for Drosophila embryo development (20), a state of high cell proliferation and organ growth when YAP transcriptional activity should be high and Hippo pathway inhibition of YAP low. In the absence of *dJub*, Hippo activity would be unrestrained, *Yki* would be inhibited, and cells would cease to proliferate and undergo apoptosis. In support of this, in mammalian cells, we were unable to RNAi deplete all three AJUBA LIM proteins, as they would undergo apoptosis whenever the third family member was depleted.

In contrast to other studies (24), we found that in mammalian cells, the AJUBA LIM proteins did not affect YAP regulation in response to mechanical signals. Since AJUBA LIM proteins regulate YAP by inhibiting activation of LATS kinases by the Hippo core kinase complex, this could be a reflection of LATS-independent regulation of YAP in response to mechanical signals (10, 15), but in other studies, morphological manipulation of single cells affected YAP regulation in a LATS-dependent manner (41). When we likewise manipulated single cells (i.e., intracellular tension [Fig. 3D and E]), AJUBA LIM proteins did not affect YAP nuclear/cytoplasmic distribution or transcriptional activity in response to changes in intracellular tension. This suggests that LATS activation in response to mechanical signals in mammalian cells, a process that is not fully understood, is not influenced by the AJUBA LIM proteins. Furthermore, if AJUBA LIM proteins affect only Hippo-dependent activation of LATS, then this result would be consistent with the model of mechanical activation of LATS independent of the Hippo core kinase complex.

During Drosophila wing development, genetic experiments have shown that *dJub* influences tension-dependent Yki-mediated wing growth (25). There, it was argued that dJub does so by recruiting Wts (LATS) to the cell junction in a tension-dependent manner (25). We also observed that the presence of LIMD1 and AJUBA influenced the recruitment of LATS1 to cell-cell junctions in a confluent mammalian epithelium (Fig. 7B), but in confluent epithelia undergoing CIP, we did not observe any inhibition of LATS kinases by AJUBA LIM proteins, nor did AJUBA LIM proteins associate with LATS kinases or the Hippo core kinase complex despite their presence at cell-cell junctions (Fig. 1 and 4). Moreover, forced recruitment of LATS2 to the plasma membrane by the mp-MOB1A mutant did not recruit LIMD1 to the plasma membrane and LATS activation was not inhibited, nor did LIMD1 associate with LATS2 or the Hippo core kinase components (Fig. 8).

In MCF10A cells, cyclic or static stretch was found to activate YAP as a result of Hippo pathway inhibition (24). There, JNK activation, downstream of a stretch signal, phosphorylated LIMD1, which enhanced its interaction with and inhibition of LATS kinase (24). In this work, RNAi depletion of LIMD1 alone was sufficient to produce an effect. In our experiments, we saw no effects when any single AJUBA LIM protein was depleted in MCF10A cells. Since AJUBA, LIMD1, and trace amounts of WTIP (the AJUBA family LIM proteins) are present in MCF10A cells, depletion of at least two (AJUBA and LIMD1) was required to observe any effect of this family of proteins upon Hippo pathway activation of LATS. Furthermore, AJUBA^−/−^ and LIMD1^−/−^ mice have minimal developmental or adult phenotypes unless stressed (26, 42, 43). We did not assess stretch as a mechanical stimulus in our studies, however. Thus, it is possible that stretch signals versus exposure to a stiff ECM activate distinct mechanotransduction pathways and that stretch-activated pathways are more sensitive to AJUBA LIM protein levels.

AJUBA LIM proteins and the Hippo core kinase components are both recruited to sites of cell contact, yet we did not detect any association between them at this site. Possibly, they are recruited to different cell contact components. AJUBA LIM proteins interact with α-catenin bound to E-cadherin at AJ (37). The ERM protein NF2 (*Mer*), which associates with the apical membrane protein Crumbs, recruits LATS (Wts) to the plasma membrane (33). AJUBA LIM proteins do not associate with Mer, Ex, or Kibra. Therefore, physical separation of AJUBA LIM proteins and Hippo core kinase components at cell-cell contacts possibly preclude an association and inhibition. Interestingly, in the absence of AJUBA and LIMD1, endogenous LATS1 was not recruited to the cell surface despite the presumed presence of NF2 (Fig. 7B). Perhaps AJUBA LIM proteins serve to facilitate delivery of LATS to NF2 at the cell surface. This observation also suggests the possibility that LATS could be activated without plasma membrane recruitment. We cannot exclude the possibility that transient recruitment of LATS to the cell surface still occurs, through NF2 for example, and that this may be enough for its activation and phosphorylation of YAP. Despite the significant change in membrane recruitment of LATS1 in confluent epithelia lacking AJUBA and LIMD1, YAP was still inactivated. Perhaps this reflects Hippo-independent or actin-mediated regulation of YAP in this setting (10, 15).

Other signals could regulate the association of AJUBA LIM proteins with LATS and the Hippo core kinase complex. MAPK can phosphorylate *dJub* and increase its association with *Wts* in S2 cells (22), for example, and in mammalian cells, overexpression of AJUBA increases MAPK activity (44). In the context of tissue repair, JNK can also phosphorylate AJUBA, and this increases its association with LATS (23). In light of these findings, AJUBA LIM proteins are possibly phosphorylated in the cytosol and not at adherens junctions. In the absence of phosphorylation, they do not interact with the Hippo core kinase complex.

In Drosophila, AJUBA LIM protein-mediated regulation of the Hippo pathway is critical for wing development. The determination of organ size, such as the fly wing and fly eye and the mammalian liver, is a cell intrinsic property involving the Hippo pathway, as shown by organ-specific transgenic models (45–50). Our work suggests that the AJUBA LIM proteins limit Hippo pathway activity only in proliferating cells. If so, then they may be critical to tune the proliferative response of cells during organ development, repair of injured tissue, and early cancer development.
